# Impact of COVID-19 lockdown on work-family balance, resilience, and burnout of physicians in Lebanon: a cross-sectional survey study

**DOI:** 10.3389/fgwh.2026.1721389

**Published:** 2026-07-14

**Authors:** Eveline Hitti, Hani Tamim, Abdo Mghames, Dima Hadid, Walaa El Sheikh, Ghada Chamandi, Lina Daouk-Öyry

**Affiliations:** 1 Department of Emergency Medicine, American University of Beirut Medical Center, Beirut, Lebanon; 2 Clinical Research Institute and Department of Internal Medicine, American University of Beirut Medical Center, Beirut, Lebanon; 3 College of Medicine, Alfaisal University, Riyadh, Saudi Arabia; 4 Health Policy PhD Program, Department of Health Research Methods, Evidence, and Impact, McMaster University, Hamilton, ON, Canada; 5 Faculty of Medicine, American University of Beirut, Beirut, Lebanon; 6 Department of Leadership and Organizational Behaviour BI Norwegian Business School, Oslo, Norway

**Keywords:** burnout, childcare, COVID-19, gender, lockdown, physicians, resilience, work-family balance

## Abstract

**Introduction:**

The COVID-19 pandemic placed significant strain on physicians. Gender inequities in medicine preceded the pandemic. This study aimed to assess the association of COVID-19 lockdown on physicians’ careers, work-life balance, resilience, and burnout from a gender perspective, in Lebanon.

**Materials and methods:**

A web-based survey was sent to all Lebanese Syndicate of Physicians members. Data was collected between August and September 2022. The survey was designed comprising five domains to assess personal characteristics, career impact, work-family intersection, resilience, and burnout.

**Results:**

A total of 165 physicians were included in the analysis. A greater percentage of women were married with children and in a non-surgical specialty. During COVID-19 lockdown, more women occupied part-time positions compared to pre-COVID-19 (29.2%vs.18.0%, *p* = 0.04), whereas there was no significant difference among men. While both men and women shifted to more remote work during COVID-19, there was no statistically significant difference by gender. Moreover, more women than men reported increased time spent on household tasks (64.0%vs.36.8%, *p* < 0.001). While the majority of both men and women reported increased parenting activities, a higher percentage of women reported solely bearing childcare responsibilities during school disruptions. Furthermore, a higher percentage of women reported relying on their extended family during childcare disruptions (30.9%vs.9.5%, *p* < 0.001), compared to men who reported relying more on their spouses (52.6%vs.9.1%, *p* < 0.001). Multivariate regression of Work-to-Family conflict and resilience scales did not show any statistically significant difference by gender, nor was there a gendered difference in burnout scores. Being married and married with children were both associated with higher resilience in physicians.

**Conclusions:**

Our study highlights the disproportionate association between COVID-19 lockdown and experiences of women physicians. Policies and initiatives to retain women physicians during crises are of paramount importance as are interventions targeting resilience in single physicians and overall burnout.

## Introduction

Gender inequities in medicine continue to exist globally. While several countries have narrowed the gender gap in medical school training (1, 2), the disparities persist and even widen at the leadership level, where women physicians remain disproportionately underrepresented (3). The gender gap is particularly striking when it comes to full-time and part-time positions, where women physicians constitute the largest portion of part-time physician workforce as many shift to part-time work looking to balance professional and personal responsibilities (4). The Coronavirus Disease of 2019 (COVID-19) pandemic brought in additional strains to both personal and professional lives of physicians in particular. Stay at home policies disrupted the childcare infrastructure leaving working parents with additional responsibilities at the home front. For physicians, COVID-19 also came with additional patient care responsibilities and challenges (5). The long-term influence of the disparities linked to these competing demands during COVID-19 on the gender gap in medicine is yet to be determined.

Several studies have explored the gender-based differences observed during the COVID-19 pandemic in physicians’ personal and professional lives. While studies on job satisfaction during COVID-19 showed lower rates of satisfaction for both men and women physicians (6, 7), studies on burnout and well-being found gender differences in physician experiences. In fact, a significant increase in burnout levels was observed among physicians in the United States (US) during the COVID-19 pandemic (8), with evidence indicating that women physicians reported higher levels of emotional exhaustion and lower levels of personal accomplishment compared to men (9). Studies have also shown that women physicians were more likely to report increased feelings of burnout when compared to men physicians, along with a decreased level of career satisfaction (10, 11). Moreover, married women physicians reported lower levels of wellbeing than married men during COVID-19 (12). This shift in levels of wellbeing raises concerns about the evolving dynamics and challenges faced by healthcare professionals, particularly when viewed through a gendered lens.

Other studies have examined the challenges linked to the COVID-19 pandemic on physicians’ careers. These studies found that women were more likely to shift to part-time work, drop academic activity, or leave the workforce (10, 13). COVID-19 pandemic highlighted the gendered division among physicians when it came to balancing between their work and family responsibilities leading to a significant disruption in work-life balance particularly among physician mothers (14). Men and women physicians both faced increased domestic and personal challenges during the pandemic, particularly in navigating their parental roles (15). Notably, women were more likely to be responsible for childcare or schooling and household tasks during the pandemic (13). In addition, more women than men were forced to take additional leaves and quit their jobs due to the increased domestic responsibilities, leaving women shouldering more of the pandemic's childcare burden (14).

While these studies have highlighted the disproportionate burden the pandemic placed on women physicians, most have been conducted in High-Income Countries (HICs). There are limited studies on how these dynamics play out in Lower-Middle Income Countries (LMICs) (16). In Lebanon, a LMIC, where the gender gap closed since 2012 at the level of medical school training (17), women continue to account for only 21.8% of the physician workforce, with even bigger under-representation in surgical specialties where women account for only 2.3% of surgeons (18). Studies that have looked at professional and personal intersection of physicians in Lebanon, found that while professional effort allocation between men and women in full-time positions was the same, women physicians bore a greater load of the parental, domestic work, and childcare responsibilities during disruptions when compared to men physicians (19). How these dynamics played out and impacted the careers of physicians during the pandemic have not yet been explored.

Our study aimed to assess the association of these unique dynamics during the COVID-19 pandemic on physicians’ careers, work-life balance, resilience, and burnout from a gender perspective, in Lebanon. Although the primary focus of this study is on the COVID-19 pandemic, it is essential to situate the findings within the broader Lebanese context, which was characterized by multiple, overlapping crises during that period (20). Lebanon has historically been recognized for having a relatively strong and efficient healthcare system, with high levels of medical expertise and service quality compared to the region (21). However, this system has demonstrated both resilience and vulnerability when confronted with sustained and compounding pressures (21). During the period under study, the COVID-19 pandemic unfolded alongside two major national crises: the economic collapse and the Beirut port explosion. The economic crisis, marked by severe currency devaluation, inflation, and the erosion of institutional capacity, significantly strained healthcare infrastructure and led to resource shortages and workforce attrition across sectors (20, 22, 23). In parallel, the Beirut port explosion in August 2020 was a catastrophic event that killed over 200 people, injured thousands, and destroyed critical infrastructure, including major hospitals. These crises created a context of prolonged instability, uncertainty, and cumulative stress, placing exceptional demands on both the healthcare system and the individuals working within it. This context suggests that the experiences captured in this study cannot be understood as responses to COVID-19 alone, but rather as embedded within a broader landscape of intersecting crises that likely intensified stressors and shaped patterns of burnout and resilience.

## Materials and methods

### Study design and setting

This study employed a cross-sectional survey design. Data were collected in Lebanon in 2022 through a web-based questionnaire, with participants retrospectively reporting on their experiences during the COVID-19 pandemic in 2020–2021. Ethical approval was obtained from the Institutional Review Board (IRB) of the American University of Beirut under the protocol number SBS-2020-0456. In the conduct of this study, the reporting adheres to the guidelines of the Strengthening the Reporting of Observational Studies in Epidemiology (STROBE) statement.

### Selection of participants

At the time of the study, a total of 4,916 emails were obtained from the syndicate of whom 60% work in Lebanon. Five emails were screened out by LimeSurvey and a total of 103 physicians chose to opt out. During the survey period, Lebanon was in the process of recovering from the COVID-19 lockdown, which had been in effect from March 15, 2020, until February 21, 2022. Participants received 3 email reminders that were effective in increasing the response rate, with each reminder being sent one week apart. All participants provided an electronic informed consent. Only participants who reported working in Lebanon during the COVID-19 period were included in the study.

### Data collection

Data were collected through a web-based survey of 51 questions that included three validated standardized scales about work-family intersection, resilience, and burnout in addition to a questionnaire section with general demographic items and career-related questions, which were specifically developed for this study and are not part of a standardized instrument. The survey was administered via Lime Survey between 23-08-2022 and 08-09-2022 to all Lebanese physicians registered in the Lebanese Syndicate of Physicians (Survey in S1 Survey). The survey was customized to the institutional setting and reviewed by a group of experts, including a statistician, the director of the Emergency Medicine Department, and a social scientist. The expert team modified the questionnaire as necessary, and a consensus was reached on the final draft, which was then piloted on 30 physicians. All feedback was consolidated in the final version of the survey.

#### Questionnaire

The questions in the questionnaire section were identified based on a review of peer-reviewed articles assessing domestic workload, childcare responsibilities, and employment changes. Here, participants reported general demographics and personal characteristics including age, gender, marital status, number and age of children if applicable, and medical specialty. The questionnaire section included questions about domestic burden and childcare responsibilities that focused on childcare status during the closure of schools and kindergartens and the conflicts in career responsibilities between the physicians and their partners during COVID-19 lockdown COVID-19 lockdown period in Lebanon extended in variable phases of stringency from March 15, 2020, till February 21, 2022, when all lockdown measures were ended. Participants were also asked about the change in their time spent on professional work, domestic tasks and parenting per week. Participants were also asked in this section questions related to their employment status, percentage of remote work and financial status pre- and during COVID-19 lockdown. Additional questions about career advancement included questions related to whether or not COVID-19 lockdown made them miss out on development and advancement opportunities.

#### Standardized scales

To evaluate the extent to which family interfered with work roles and responsibilities (family-to-work conflict) (FWC) and work interfered with family roles and responsibilities (work-to-family conflict) (WFC), participants were asked to complete the Work-Family Conflict Scale (WAFCS) (24). The WAFCS consists of 10 statements. Respondents rated their level of agreement with each item on a 7-point Likert scale from 1 (very strongly disagree) to 7 (very strongly agree). Scores range from 5 to 35 points. The WAFCS has strong concurrent validity and demonstrates high internal consistency (>0.90) and reliability (24).

To assess the physicians’ ability to bounce back from adversity, we used the Brief Resilience Scale (BRS) (26). The BRS consists of six statements that measure an individual’s ability to cope with stress and recover from setbacks, and each statement is rated on a different scale. Participants were asked to rate their level of agreement on 6 statements using a 5-point Likert scale: statements 1, 3 and 5 are rated from 1 (strongly disagree) till 5 (strongly agree), whereas statements 2, 4 and 6 are rated from 5 (strongly disagree) till 1 (strongly agree). To calculate the BRS score, the scores of all six statements were added up in order to obtain a total score ranging between 6 and 30 and then divided by the total number of questions answered, with higher scores indicating higher resilience (26).

To assess physicians’ burnout during COVID-19, we used the 9-item abbreviated Maslach Burnout Inventory (aMBI) (27). The MBI consists of three subscales that measure different dimensions of burnout (27): emotional exhaustion (items 1, 6 and 9), depersonalization (items 2, 5 and 8), and personal accomplishment (items 3, 4 and 7). Each item is rated from 0 (never) to 6 (every day) and each subscale is scored separately. For personal accomplishment subscale, a score equal or greater than 15 indicates a low burnout, 13–14 indicates a moderate burnout and a score equal or lower than 12 indicates a high burnout. For the depersonalization subscale, a score equal or lower than 3 indicates a low burnout, 4–6 indicates a moderate burnout and a score equal or greater than 7 indicates a high burnout. For the emotional exhaustion subscale, a score equal or lower than 6 indicates a low burnout, 7–10 indicates a moderate burnout and a score equal or greater than 11 indicates a high burnout.

### Statistical analysis

The data was analyzed and processed using the SPSS version 28.0 (IBM Corp., Armonk, N.Y., USA). Categorical variables were summarized by calculating the number and percentages, whereas the continuous variables were summarized as mean and standard deviation (±SD). Bivariate comparisons were carried out by using the Chi-square test for the categorical variables in order to assess the impact of COVID-19 pandemic on careers, and the independent Student's t-test for the continuous ones. To identify factors associated with work-family conflict and resilience of physicians, we constructed a series of multivariate linear regression models with the following participant characteristics: age, gender, medical specialty, marital status with children, employment status during COVID-19, employment setting during COVID-19, financial status during COVID-19, and percentage of remote work during COVID-19. Results were presented as beta (*β*) and 95% confidence interval (CI). Statistical significance was considered at the 0.05 level.

## Results

A total of 195 participants completed the survey. Only 165 physicians were residing in Lebanon during COVID-19 lockdown, and were thereby included in the final analysis, achieving a response rate of 6.6%.

Table 1 represents the demographic and professional characteristics of the participants, by gender. Women comprised 53.9% of the participants with men physicians being relatively older than their women counterparts (mean age 39.9 ± 9.6 vs. 36.1 ± 6.0, *p* = 0.003). Among men physicians, 46.1% were aged ≤ 36 years, whereas 58.4% of women physicians were aged ≤ 36 (*p* = 0.01). In addition, a greater percentage of women physicians were married with children (60.7% vs. 55.3%, *p* = 0.46). Moreover, surveyed women physicians were more in a non-surgical specialty (88.8% vs. 71.1%, *p* = 0.004). Women worked more in private practice settings, while a higher percentage of men practiced in academic medical centers (44.3% vs. 28.0% and 57.3% vs. 38.6% respectively, *p* = 0.048).

**Table 1:** Demographic and professional characteristics, by gender.

Demographics	Details	Gender		
		Men (*n* = 76)	Women (*n* = 89)	*p*-value
Age	Mean (SD)	39.9 (9.6)	36.1 (6.0)	**0.003**
	≤ 36 y.o.	35 (46.1%)	52 (58.4%)	**0.01**
	36–50 y.o.	30 (39.5%)	35 (39.3%)	
	≥ 51y.o.	11 (14.5%)	2 (2.2%)	
Marital status	Single	25 (32.9%)	20 (22.5%)	0.31
	Married	49 (64.5%)	65 (73.0%)	
	Other (Divorced, Widower, Engaged)	2 (2.6%)	4 (4.5%)	
Children	Yes	42 (55.3%)	55 (61.8%)	0.40
Marital status + Children	Single	27 (35.5%)	24 (27.0%)	0.46
	Married	7 (9.2%)	11 (12.4%)	
	Married with children	42 (55.3%)	54 (60.7%)	
Number of children	1	10 (23.8%)	19 (34.5%)	0.47
	2	22 (52.4%)	23 (41.8%)	
	≥ 3	10 (23.8%)	13 (23.6%)	
Age of the youngest child	Mean (SD)	6.5 (7.8)	4.7 (5.9)	0.198
	< 5 y.o.	27 (64.3%)	43 (78.2%)	0.39
	5–12 y.o.	9 (21.4%)	9 (16.4%)	
	12–18 y.o.	2 (4.8%)	1 (1.8%)	
	≥ 18 y.o.	4 (9.5%)	2 (3.6%)	
What facility do/does your child/children attend?^a^^,^^b^	Nursery (for children younger than three years)	14 (33.3%)	20 (36.4%)	0.76
	School	24 (57.1%)	39 (70.9%)	0.16
	University	6 (14.3%)	3 (5.5%)	0.17
	Homeschool	2 (4.8%)	2 (3.6%)	1.00
	Child is no longer a student	5 (11.9%)	1 (1.8%)	0.08
Specialty	Non-Surgical	54 (71.1%)	79 (88.8%)	**0.004**
	Surgical	22 (28.9%)	10 (11.2%)	
Employment setting	Private practice	21 (28.0%)	39 (44.3%)	**0.048**
	Academic medical center	43 (57.3%)	34 (38.6%)	
	Other	11 (14.7%)	15 (17.0%)	

The bold values correspond to signficant values <0.05.

a Multiple answers were allowed.

b Among parents with children.

Table 2 represents the impact of COVID-19 lockdown on careers. During COVID-19 lockdown, more women occupied part-time positions compared to pre-COVID-19 (29.2% vs. 18.0%, *p* = 0.04), whereas there was no significant difference in employment status for men. While both men and women experienced significant worsening of their financial status during COVID-19 (22.4% and 14.6% vs. 10.5% and 7.9%, respectively, *p* < 0.001), there was no statistically significant difference by gender. Moreover, both men and women physicians shifted to more remote work during COVID-19 lockdown (18.7% and 21.6% vs. 6.7% and 4.5%, respectively), however, there was no statistically significant gendered difference.

**Table 2:** Impact of COVID-19 lockdown on careers.

Variables	Details	Pre-COVID-19		*p*-value	During COVID-19		*p*-value		
		Men (*n* = 76)	Women (*n* = 89)		Men (*n* = 76)	Women (*n* = 89)		Men (*n* = 76)	Women (*n* = 89)
								*p*-value (pre/during COVID-19)	*p*-value (pre/during COVID-19)
Employment status	Full-time	56 (73.7%)	72 (80.9%)	0.25	54 (71.1%)	59 (66.3%)	0.54	0.15	**0.04**
	Part-time	16 (21.1%)	16 (18.0%)		17 (22.4%)	26 (29.2%)			
	Not employed	4 (5.3%)	1 (1.1%)		5 (6.6%)	4 (4.5%)			
Financial status	Sometimes struggle to make ends meet	8 (10.5%)	7 (7.9%)	0.79	17 (22.4%)	13 (14.6%)	0.43	**<0.001**	**<0.001**
	Just about enough to get by	26 (34.2%)	29 (32.6%)		41 (53.9%)	52 (58.4%)			
	Make more than I need to live well	42 (55.3%)	53 (59.6%)		18 (23.7%)	24 (27.0%)			
Percentage of remote work	0–25%	65 (86.7%)	70 (79.5%)	0.17	45 (60.0%)	44 (50.0%)	0.42	**<0.001**	**<0.001**
	26–50%	5 (6.7%)	14 (15.9%)		16 (21.3%)	25 (28.4%)			
	51–100%	5 (6.7%)	4 (4.5%)		14 (18.7%)	19 (21.6%)			

The bold values correspond to signficant values <0.05.

Table 3 represents the work-life intersection and perception of COVID-19 lockdown towards impact on career. Our results showed that more women than men reported a decrease in their time spent on professional work, during COVID-19 lockdown (51.1% vs. 36.0%, *p* = 0.13). Moreover, more women than men reported increased time spent on household tasks (64.0% vs. 36.8%, *p* = <0.001). While the majority of both men (57.1%) and women (61.8%) reported increased parenting activities during COVID-19 lockdown, a higher percentage of women reported solely bearing childcare responsibilities during school disruptions (32.7% vs. 2.6%, *p* < 0.001). Furthermore, a higher percentage of women reported relying on their extended family during childcare disruptions (30.9% vs. 9.5%, *p* < 0.001), compared to men who reported relying on their spouses (52.6% vs. 9.1%, *p* < 0.001). Moreover, there was no statistically significant difference between men and women when it came to missing out on development and advancement opportunities during COVID-19 lockdown (73.3% and 68.0% vs. 85.2% and 78.4%, *p* = 0.06 and 0.13, respectively).

**Table 3:** Work life-intersection and perception of COVID-19 lockdown towards impact on careers.

Characteristics	Sub-characteristics	Details	Gender		*p*-value
			Men (*n* = 76)	Women (*n* = 89)	
Effort-allocation (Time spent on/week change)	Professional work	Increased	37 (49.3%)	31 (35.2%)	0.13
		Stayed the Same	11 (14.7%)	12 (13.6%)	
		Decreased	27 (36.0%)	45 (51.1%)	
	Household tasks	Increased	28 (36.8%)	57 (64.0%)	**<0.001**
		Stayed the Same	35 (46.1%)	28 (31.5%)	
		Decreased	13 (17.1%)	4 (4.5%)	
	Parenting^a^	Increased	24 (57.1%)	34 (61.8%)	0.86
		Stayed the Same	13 (31.0%)	16 (29.1%)	
		Decreased	5 (11.9%)	5 (9.1%)	
Children-related^a^	During COVID-19 lockdown, did your child's day care or school get impacted?	Yes	30 (71.4%)	52 (94.5%)	**0.002**
	Who was mainly responsible for childcare due to the closure of schools and kindergartens?	Mainly me	1 (2.6%)	18 (32.7%)	**<0.001**
		Mainly my spouse	20 (52.6%)	5 (9.1%)	
		Me and my spouse equally	12 (31.6%)	13 (23.6%)	
		Extended family	4 (9.5%)	17 (30.9%)	
		Child does not need care	4 (9.5%)	0 (0.0%)	
		Other	1 (2.4%)	2 (3.6%)	
Work-home conflicts	During the COVID-19 lockdown, did you experience increased conflict between you and your spouse's/partner's career responsibilities?	Yes	17 (37.8%)	21 (34.4%)	0.72
	During the COVID-19 lockdown, whose career took priority when conflict arose between you and your spouse's/partner's career responsibilities	My Spouse's/Partner's Career Took Priority	5 (11.1%)	8 (13.1%)	0.93
		My Career Took Priority	13 (28.9%)	16 (26.2%)	
		We Shared it Relatively Equally	27 (60.0%)	37 (60.7%)	
Work opportunities	During the COVID-19 lockdown in Lebanon, did you miss out on development opportunities?	Yes	55 (73.3%)	75 (85.2%)	0.06
	During the COVID-19 lockdown in Lebanon, did you miss out on advancement opportunities?	Yes	51 (68.0%)	69 (78.4%)	0.13

The bold values correspond to signficant values <0.05.

a Men (*n* = 42), Women (*n* = 55).

Table 4 represents the multivariate linear regression models of Work-to-Family Conflict and Family-to-Work Conflict among physicians. Higher age was associated with lower work-to-family conflict (*β* = −0,21; 95% CI, −0.38 to −0.03; *p* = 0.02). Women reported lower work-to-family conflict (*β* = −0,87; 95% CI, −3.37–1.62; *p* = 0.49) and a greater family-to-work conflict (*β* = 0.86; 95% CI, −1.18–2.91; *p* = 0.41). Although there was no statistically significant difference, physicians with a surgical specialty reported a greater family-to-work conflict (*β* = 0.48; 95% CI, −2.06–3.02; *p* = 0.71), and married physicians with children reported a lower work-to-family conflict (*β* = −0,59; 95% CI, −3.37–2.18; *p* = 0.67) and a greater family-to-work conflict (*β* = 1.50; 95% CI, −0.78–3.78; *p* = 0.19). Furthermore, physicians working 51%–100% of their time remotely, reported a lower work-to-family conflict (*β* = −2,58; 95% CI, −5.55–0.39; *p* = 0.09).

**Table 4:** Multivariate linear regression models of work-to-family conflict and family-to-work conflict among physicians.

Characteristic	Details	Work-to-family conflict	*p*- value	Family-to-work conflict	*p*-value
		*β* (95% CI)		β (95% CI)	
Age		−0.21 (−0.38, −0.03)	**0.02**	−0.09 (−0.23, 0.06)	0.23
Gender	Men	1 [Reference]	NA	1 [Reference]	NA
	Women	−0.87 (−3.37, 1.62)	0.49	0.86 (−1.18, 2.91)	0.41
Specialty	Non-Surgical	1 [Reference]	NA	1 [Reference]	NA
	Surgical	0.01 (−3.09, 3.10)	0.995	0.48 (−2.06, 3.02)	0.71
Marital status with children	Single	1 [Reference]	NA	1 [Reference]	NA
	Married	−1.19 (−5.28, 2.90)	0.57	0.68 (−2.68, 4.03)	0.69
	Married with children	−0.59 (−3.37, 2.18)	0.67	1.50 (−0.78, 3.78)	0.19
Employment status during COVID	Full-time	1 [Reference]	NA	1 [Reference]	NA
	Part-time	0.35 (−2.59, 3.29)	0.81	1.72 (−0.69, 4.13)	0.16
	Not Employed	−0.03 (−6.39, 6.33)	0.99	0.04 (−5.18, 5.26)	0.99
Employment setting during COVID	Private Practice	1 [Reference]	NA	1 [Reference]	NA
	Academic Medical Center	2.40 (−0.41, 5.20)	0.09	1.07 (−1.23, 3.37)	0.36
	Other	0.61 (−2.84, 4.07)	0.73	−0.58 (−3.41, 2.26)	0.69
Financial status during COVID	Sometimes Struggle to Make Ends Meet	1 [Reference]	NA	1 [Reference]	NA
	Just About Enough to Get by	−1.30 (−4.46, 1.86)	0.42	−1.60 (−4.19, 1.00)	0.23
	More than I Need to Live Well	−1.67 (−5.30, 1.97)	0.37	−1.40 (−4.38, 1.58)	0.35
Percentage of remote work during COVID	0–25%	1 [Reference]	NA	1 [Reference]	NA
	26–50%	0.93 (−1.82, 3.68)	0.51	1.1 (−1.13, 3.32)	0.33
	51–100%	−2.58 (−5.55, 0.39)	0.09	0.1 (−2.30, 2.50)	0.93

The bold values correspond to signficant values <0.05.

Table 5 represents the multivariate linear regression models of resilience scale among physicians. Women reported lower resilience when compared to men (*β* = −0.15; 95% CI, −0.36–0.07; *p* = 0.18), whereas married physicians and those with children reported greater resilience when compared to single physicians (*β* = 0,47; 95% CI, 0.12–0.82; *p* = 0.008 and *β* = 0.26; 95% CI, 0.02–0.49; *p* = 0.034, respectively). In addition, part-time physicians reported lower resilience when compared to full-timers (*β* = −0,32; 95% CI, −0.57 to −0.07; *p* = 0.013). Furthermore, physicians making more than they need to live well reported higher resilience when compared to those who sometimes struggle to make ends meet (*β* = 0,29; 95% CI, −0.02–0.6; *p* = 0.07).

**Table 5:** Multivariate linear regression models of resilience scale Among physicians.

Characteristic	Details	Resilience Scale	*p*- value
		β (95% CI)	
Age		0.01 (−0.01, −0.02)	0.44
Gender	Men	1 [Reference]	NA
	Women	−0.15 (−0.36, 0.07)	0.18
Specialty	Non-Surgical	1 [Reference]	NA
	Surgical	0.04 (−0.23, 0.30)	0.79
Marital status with children	Single	1 [Reference]	NA
	Married	0.47 (0.12, 0.82)	**0.008**
	Married with children	0.26 (0.02, 0.49)	**0.034**
Employment status during COVID	Full-time	1 [Reference]	NA
	Part-time	−0.32 (−0.57, −0.07)	**0.013**
	Not Employed	−0.4 (−0.94, 0.14)	0.15
Employment setting during COVID	Private Practice	1 [Reference]	NA
	Academic Medical Center	−0.02 (−0.26, 0.22)	0.84
	Other	−0.30 (−0.60, −0.01)	**0.043**
Financial status during COVID	Sometimes Struggle to Make Ends Meet	1 [Reference]	NA
	Just About Enough to Get by	0.08 (−0.19, 0.35)	0.55
	More than I Need to Live Well	0.29 (−0.02, 0.6)	0.07
Percentage of remote work during COVID	0–25%	1 [Reference]	NA
	26–50%	−0.18 (−0.43, 0.07)	0.16
	51–100%	0.17 (−0.11, 0.45)	0.23

The bold values correspond to signficant values <0.05.

Table 6 represents the burnout score, by gender. The scores for aMBI were computed for each subscale. Higher scores indicate higher levels of burnout, except for personal accomplishment, where lower scores indicate greater levels of burnout. Results for personal accomplishment were clustered at the high and low ends of the scale, with (43.6%) of participants reporting low levels and (38.7%) reporting high levels, while only (17.8%) fell within the moderate range. Regarding gender differences, a higher percentage of women reported greater levels of emotional exhaustion compared to their men counterparts (34.1% vs. 28.0%, *p* = 0.38) although results were non-significant.

**Table 6:** Burnout score, by gender.

	Score	N	Men	Women	*p*-value
Personal Accomplishment	Low	71 (43.6%)	33 (44.0%)	38 (43.2%)	0.46
	Moderate	29 (17.8%)	16 (21.3%)	13 (14.8%)	
	High	63 (38.7%)	26 (34.7%)	37 (42.0%)	
Depersonalization	Low	107 (65.6%)	44 (58.7%)	63 (71.6%)	0.22
	Moderate	33 (20.2%)	18 (24.0%)	15 (17.0%)	
	High	23 (14.1%)	13 (17.3%)	10 (11.4%)	
Emotional Exhaustion	Low	60 (36.8%)	26 (34.7%)	34 (38.6%)	0.38
	Moderate	52 (31.9%)	28 (37.3%)	24 (27.3%)	
	High	51 (31.3)	21 (28.0%)	30 (34.1%)	

## Discussion

Our study assessed the association of COVID-19 lockdown on physicians’ careers, work-life balance, resilience, and burnout from a gender perspective, in Lebanon. During COVID-19 lockdown, more women reported working in part-time positions compared to pre-COVID-19. Even though this shift to part-time was not experienced by men physicians, a higher percentage of men physicians faced challenges in making ends meet during COVID-19 pandemic compared to their women counterparts. On the domestic front, more women than men reported increased time spent on household tasks. While the majority of both men and women reported increased parenting activities during COVID-19 lockdown, a higher percentage of women reported solely bearing childcare responsibilities during school disruptions. During these disruptions, a higher percentage of women reported relying on extended family, compared to men who reported relying more on their spouses. Multivariate linear regression of WAFCS and resilience scale did not show any statistically significant difference by gender. Our study also revealed that results of personal accomplishment among physicians were concentrated at both the high and low ends of the scale, and there were no gendered differences in burnout scores.

Studies looking at physician workload during COVID-19 pandemic showed an overall decrease in physicians’ working hours. This is likely attributable to the drop-in health service utilization that was observed globally (27–29). Our study revealed that more women (51.1%) than men (36.0%) reported a decrease in their time spent on professional work. Additionally, more women in our study reported shifting to part-time work during the pandemic compared to men physicians. This aligns with other studies investigating shifts in employment status among physicians during COVID-19, which found that more women physicians moved to part-time positions than men because of increased childcare responsibilities, schooling demands, and household tasks (13, 30). In spite of women scaling back during the pandemic, a smaller percentage of women compared to men reported facing financial challenges during the pandemic. This could be explained by women physicians more likely being in dual career relationships compared to men physicians (19), although we did not explore this specifically in our study.

While parenting responsibilities increased for both men and women physicians during COVID-19 in our study, a greater percentage of women reported increases in household responsibility and bearing primary childcare responsibility during disruptions in school or childcare than men physicians. These disruptions were protracted in Lebanon where the government adopted one of the highest stringency approaches towards COVID-19 globally, implementing strict and early lockdowns of schools, childcare services, and universities within days of the first identified case. This occurred amidst the collapse of the banking sector in Lebanon, that had pushed the country into an economic crisis, leaving no governmental resources to support families through the interruptions of childcare. Our data depicted a significant decline in the financial status of all physicians due to COVID-19 lockdown (*p* < 0.001). Moreover, lack of state-level support, aggravated by the financial crisis, placed a burden on personal and extended family networks (25). Given the growing body of evidence on “domestic tethers” and how inequities in domestic care impact career choices for physicians (19, 31), the increasing household and parental responsibilities for women physicians during COVID-19 could explain the greater shift to part-time work reported by women physicians during COVID-19.

In spite of the increase in both household and parenting work reported by women physicians in our study during COVID-19, there were no significant gendered difference regarding WFC and FWC scales, in contrast to other studies where mothers reported higher scores for both WFC and FWC (13). This could be because of the greater drop in work hours reported by women in our study, and therefore fewer demands from their professional responsibilities. Another explanation could be related to the availability of an extended family support in our study context, which women reported relying heavily on during disruptions of childcare. The extended family support structure was a distinctive finding in our study compared to studies done in Canada where facility based childcare services were more common (32). In fact, in our study, older age was the only variable that had a statistically significant association with lower WFC, presumably because of fewer dependents with older age. Other variables that were associated with lower WFC included working remotely and financial security (reporting that they had “more than I need to live well”), although these were not found to be statistically significant. On the other hand, working at academic medical centers, while not statistically significant, was associated with a higher WFC. This may be related to the heavy burden of COVID-19 care shouldered by physicians at academic medical centers in Lebanon which were the governmentally designated COVID-19 care facilities along with the public hospitals.

We did not find significant gender differences in resilience. This is in contrast to other studies that found lower resilience levels among single women healthcare workers, compared with their men coworkers (12). Furthermore, we found that married physicians reported higher resilience scores compared to single physicians in general. Married individuals may experience higher social support than single physicians, which is a key variable associated with resilience (33, 39). The association of marriage with higher resilience persisted even in those married with children. This is in line with other studies that looked at physician resilience during COVID-19 (34, 40). In fact, studies that explored the relationship between parenthood and resilience found a positive association with notably more positive emotions in parents compared to nonparents as well as sense of meaningfulness (35, 41). A possible explanation for these findings lies in differences in life circumstances. For example, married physicians with children may be more likely to rely on broader social support networks, which can serve as an important buffer in times of crisis (33). They also tend to be older and at more advanced stages of their careers, often benefiting from greater professional stability and accumulated experience. Prior research shows that sociodemographic traits are key predictors of crisis outcomes with younger age often linked to higher anxiety (36–38). This suggests that the higher resilience observed in married or older physicians may be driven by the accumulation of psychological and material resources over time. Thus, in spite of the competing professional and personal demands of parenthood, our study suggests that even in physicians amidst a pandemic, the positive elements of parenthood outweigh the negative stressors of care burden when it comes to resilience.

While we did not find any gender-based differences in burnout, our results reveal a substantial overall burden affecting the entire physician population. There are some interesting cross-cultural differences in burnout between our physician group and burnout rates amongst physicians in other settings during the pandemic. Our study revealed a notably higher prevalence of low personal accomplishment (43.6%) compared to findings from Canada and China (22% and 20.1% respectively) (9, 39, 42). This suggests that a significant proportion of physicians in our population regardless of gender may be experiencing reduced job satisfaction and a sense of inefficacy, which are critical components of burnout as it relates to personal accomplishment. The findings are likely to be shaped by the unique and compounding contextual realities of Lebanon. Unlike settings affected primarily by COVID-19, Lebanon was simultaneously experiencing a severe economic collapse exacerbated by the Beirut port blast that devastated the city and its inhabitants (25). These intersecting crises may have intensified stressors on physicians, amplifying burnout levels beyond what might be observed in more stable contexts. Further studies are needed to better understand the contextual contributors to this finding.

This study underscores the gendered disparities in the associated with the COVID-19 lockdown on physicians and the fragility of the advances in closing the gender gap within the physician workforce. Women physician careers are more vulnerable to events that cause disruptions in the professional-family and care infrastructure. Building in more workforce resilience is important to retaining the talent, diversity, and engagement of the entire physician body, especially in times of crisis. This requires an all-society response. Addressing the disparities in domestic work, which were exacerbated during the pandemic across all contexts, is key to any initiative that aims to address the leakage of women out of the workforce. Governmental and organizational regulations around parental/family leave also play an important role, as do facility based childcare services that can support parents during disruptions of care. These policies and care structures need to be made available for both men and women to ensure progress made in closing the gender gap during times of stability, are not lost during times of crisis.

Our study also highlights the issue of resilience and burn-out of physicians in time of competing demands. While organizational efforts to address retention of women physicians are important, these need to also be coupled with support for single physicians who seem to have lower resilience and may benefit from different types of peer support. Additionally, understanding burnout especially as it relates to drivers of low personal accomplishment are important areas that need further exploration. Beyond gendered differences, the levels of burnout, resilience, and work-family conflict reported across the entire sample are substantively significant. In the Lebanese context, these findings suggest that the magnitude of the national crisis creates a universal baseline of strain affecting all physicians. Documenting these findings in an underrepresented setting is essential for informing the comprehensive policy interventions required to support the medical workforce.

## Limitations

This study has some limitations that need to be acknowledged. First, the small sample size of the study limited the power of statistical analysis. The low response rate (6.6%) was a significant limitation. This potential non-response bias limits the generalizability of our findings to the broader population of Lebanese physicians. The retrospective nature of this survey, conducted in 2022 to assess experiences during the 2020 lockdown, introduces potential recall bias. In addition, the COVID-19 lockdown period overlapped with economic and political turmoil in Lebanon that could have impacted physician's experiences. These however were experienced by both men and women, so the gendered analysis from a crisis perspective remains valid.

## Conclusions

This study highlights that women physicians were disproportionately affected in terms of increased childcare responsibilities and shifts to part-time work during the COVID-19 lockdown compared to men. It also draws attention to high overall burden of burnout associated with low personal accomplishment, affecting nearly half of the surveyed physicians during the pandemic as well as the need to support single physicians who score lower on resilience scales than married physicians. Ultimately, these findings were interpreted within the context of Lebanon's multiple overlapping crises, which most likely augmented the professional and psychological challenges faced by physicians during this period.

## Data Availability

Data are available upon reasonable request. The datasets generated and/or analysed during the current study are not publicly available but are available from the corresponding author on reasonable request.
