# Supplementary material for: Impact of COVID-19 lockdown on work-family balance, resilience, and burnout of physicians in Lebanon: a cross-sectional survey study
Source: Front Glob Womens Health. 2026 Jul 14;7:1721389. doi: 10.3389/fgwh.2026.1721389 (PMC13408004; doi:10.3389/fgwh.2026.1721389)
Supplement: Supplementary file 1 [file Datasheet1.pdf]

Impact of Covid-19 lockdown on Work-family balance, Burnout and Resilience of Lebanese physicians

Questionnaire

I. Personal characteristics

1. Age:
2. Gender
    a) Male
    b) Female

3. What is your marital status?
    a) Married
    b) Single
    c) Divorced
    d) Widowed or widower

4. Do you have children?
    a) Yes
    b) No

5. If yes to #6, how many children do you have:
6. Please specify the age(s) of your child/children:

7. What is your specialty?

a) Emergency Medicine
b) Surgery
c) Radiation oncology
d) Psychiatry
e) Physical therapy
f) Pediatrics and adolescent medicine
g) Pathology and laboratory medicine
h) Otolaryngology and Head and Neck Surgery
i) Ophthalmology
j) Neurology
k) Obstetrics/Gynecology
l) Dermatology
m) Diagnostic Radiology
n) Family Medicine
o) Internal Medicine
p) Anesthesiology
q) Ambulatory Services
r) Other (please specify):

<u>Exposure to stressors:</u>

8. **Please rate the following from 1 (least significant) to 5 (Most significant).** ***If the statement is not applicable please fill in '0'.***

American University of Beirut
Institutional Review Board
08 August 2022
APPROVED

9.

	COVID-19	Financial/economic collapse	Beirut port explosion
**My Family related pressures have increased because of this stressor**			
**My work related pressures have increased because of this stressor**			
**My mental health was impacted by this stressor**			

10. Comparing the period during COVID-19 lockdown with the period before it, please answer the following questions:

	Increased significantly	Increased	Stayed the same	Decreased	Decreased significantly
**How did the time you spent on professional work per week change?**					
**How did the time you personally spent on domestic tasks (e.g., cleaning, cooking, laundry, etc.) per week change?**					
**How did the time you personally spent on parenting**					

American University of Beirut
Institutional Review Board
08 August 2022
APPROVED

**tasks per week change?**					

II. Work-family Conflict Scale

11. **Please rate how much you agree or disagree with the following statements:**

	**Very strongly disagree 1**	**2**	**3**	**4**	**5**	**6**	**Very strongly agree 7**
**My work prevents me from spending sufficient quality time with my family**							
**There is no time left at the end of the day to do the things I'd like at home (e.g., chores and leisure activities)**							
**My family misses out because of my work commitments**							
**My work has a negative impact on my family life**							
**Working often makes me irritable or short tempered at home**							
**My work performance suffers because of my personal and family commitments**							
**Family related concerns or responsibilities often distract me at work**							
**If I did not have a family I'd be a better employee**							

American University of Beirut
Institutional Review Board
08 August 2022
APPROVED

**My family has a negative impact on my day to day work duties**							
**It is difficult to concentrate at work because I am exhausted by family responsibilities.**							

12. What facility do/does your child/children attend (Tick all that applies)?
    a) Nursery (for children younger than three years)
    b) School
    c) University
    d) Homeschool
    e) Child is no longer a student

13. During COVID-19 lockdown, did your child's day care or school get impacted?
    a) Yes
    b) No

14. Who was mainly responsible for childcare due to the closure of schools and kindergartens?
    a) Mainly me
    b) Mainly my spouse
    c) Me and my spouse equally
    d) Family
    e) Child does not need care
    f) Other, please specify:

15. During the COVID-19 lockdown, did you experience increased conflict between you and your spouse's/partner's career responsibilities:
    a) Yes
    b) No
16. During the COVID-19 lockdown, whose career took priority when conflict arose between your and your spouse's/partner's career responsibilities:
    a) My spouse's/partner's took priority
    b) My career took priority
    c) We shared it relatively equally

III. Job Impact

17. What was your employment status during the following periods:

American University of Beirut
Institutional Review Board
08 August 2022
APPROVED

	Pre-COVID-19 lockdown	During COVID-19 lockdown
**Employment status**	a) Full-time<br>b) Part-time<br>c) Not employed	a) Full-time<br>b) Part-time<br>c) Not employed

What was your employment setting during the following period:

	Pre-COVID-19 lockdown	During COVID-19 lockdown
**Employment setting**	a) Private Practice<br>b) Academic medical center<br>c) Other, please specify:	d) Private Practice<br>e) Academic medical center<br>f) Other, please specify:

What was the country of your primary practice setting during the following periods

	Pre-COVID-19 lockdown	During COVID-19 lockdown
**Country of primary practice setting**		

What percentage of your work was remote during the following period

	Pre-COVID-19 lockdown	During COVID-19 lockdown
**What percentage of your work was remote?**		

What was your financial status/income sufficiency during the following period?

	Pre-COVID-19 lockdown	During COVID-19 lockdown
**Financial status/income sufficiency**	a) Sometimes struggle to make ends meet<br>b) Just about enough to get by<br>c) More than I need to live well	d) Sometimes struggle to make ends meet<br>e) Just about enough to get by<br>f) More than I need to live well

American University of Beirut
Institutional Review Board
08 August 2022
APPROVED

19. During the COVID-19 lockdown in Lebanon, did you miss out on development opportunities?

a) Yes
b) No

19. During the COVID-19 lockdown in Lebanon, did you miss out on advancement opportunities?

a) Yes
b) No

20. Since COVID-19 began, did you change your job?

a) Yes, please specify:
b) No

Resilience Scale

Please rate how much you agree or disagree with the following statements:

	**Strongly disagree**	**Disagree**	**Neutral**	**Agree**	**Strongly agree**
I tend to bounce back quickly after hard times					
I have a hard time making it through stressful events					
It does not take me long to recover from a stressful event					
It is hard for me to snap back when something bad happens					
I usually come through difficult times with little trouble					
I tend to take a long time to get over set-backs in my life					

IV. Work-related Burnout Scale

**How often:**	**Never**<br>**0**	**A few times a year**<br>**1**	**Once a month or less**<br>**2**	**A few times a month**<br>**3**	**Once a week**<br>**4**	**A few times a week**<br>**5**	**Every day**<br>**6**
I deal very effectively with the problems of my patients.							
I feel I treat some patients as if they were impersonal objects							
I feel emotionally drained from my work.							

American University of Beirut
Institutional Review Board
08 August 2022
APPROVED

I feel fatigued when I get up in the morning and have to face another day on the job.							
I've become more callous towards people since I took this job.							
I feel I'm positively influencing other people's lives through my work.							
Working with people all day is really a strain for me.							
I don't really care what happens to some patients.							
I feel exhilarated after working closely with my patients.							

Willingness to be interviewed in a follow-up interview

Are you willing to participate in a 30-min follow-up interview? If yes please include an email where you can be reached. If no, please leave this space blank.

American University of Beirut
Institutional Review Board
08 August 2022
APPROVED
